# CXCL12/CXCR4 signaling counteracts docetaxel-induced microtubule stabilization via p21-activated kinase 4-dependent activation of LIM domain kinase 1

**DOI:** 10.18632/oncotarget.2571

**Published:** 2014-11-11

**Authors:** Arun Bhardwaj, Sanjeev K. Srivastava, Seema Singh, Sumit Arora, Nikhil Tyagi, Joel Andrews, Steven McClellan, James E. Carter, Ajay P. Singh

**Affiliations:** ^1^ Department of Oncologic Sciences, Mitchell Cancer Institute, University of South Alabama, Mobile, Alabama, USA; ^2^ Department of Pathology, College of Medicine, University of South Alabama, Mobile, Alabama, USA; ^3^ Department of Biochemistry and Molecular Biology, College of Medicine, University of South Alabama, Mobile, Alabama, USA

**Keywords:** CXCL12/CXCR4, docetaxel, microtubules, PAK4, LIMK1

## Abstract

Emerging data highlight the significance of chemokine (C-X-C motif) ligand 12/chemokine (C-X-C motif) receptor 4 (CXCL12/CXCR4) signaling axis in the chemoresistance of several malignancies, including prostate cancer (PCa); however, underlying mechanisms remain largely elusive. Here, we demonstrate that CXCL12 treatment rescues the PCa cells from docetaxel (DTX)-induced toxicity by overriding its effect on cell cycle (G_2_/M phase arrest). We further demonstrate that the chemoprotective effect of CXCL12 is abolished upon pharmacological inhibition or RNA interference-mediated silencing of CXCR4. Moreover, microtubule stabilization caused by DTX is suppressed in CXCL12-stimulated PCa cells as revealed by immunofluorescence and immunoblot analyses. The effect of CXCL12 on microtubule stabilization is abrogated when PCa cells are pre-treated with a CXCR4 antagonist. In additional studies, we show that the chemoprotective action of CXCL12/CXCR4 signaling is mediated by p21-activated kinase 4 (PAK4)-dependent activation of Lim domain kinase 1 (LIMK1), and inhibition of either PAK4 or LIMK1 leads to re-sensitization of PCa cells to DTX-induced tubulin polymerization and cellular toxicity even in the presence of CXCL12. Altogether, our findings uncover a novel mechanism underlying CXCL12/CXCR4 signaling-induced PCa chemoresistance and suggest that targeting of this signaling axis or its downstream effector pathway could lead to therapeutic enhancement of DTX.

## INTRODUCTION

Despite enormous scientific advancement over the past few decades, prostate cancer (PCa) still remains the second leading cause of cancer-related death in males in the United States [1]. The American Cancer Society estimates that nearly 233,000 new cases of PCa will be diagnosed and that about 29,480 people will die of this disease this year in the United States [1]. The first line of therapy for metastatic PCa is chemical or medical castration; however, most tumors relapse in castration-resistant (CR) form after an initial response [2, 3]. Standard optional treatment for such patients with symptomatic metastatic CR PCa is docetaxel (DTX)-based chemotherapy, but in most cases it offers survival advantage only for a short period of time (~3 months) due to chemoresistance [4, 5]. Therefore, further research is required to understand the molecular mechanisms underlying DTX-resistance in PCa, which could be helpful in formulating alternative and superior therapeutic strategies.

DTX is a member of the ‘taxane’ group of chemotherapeutic agents. It binds to the β-tubulin present in the microtubules (MTs), causing mitotic arrest and subsequent apoptosis [6]. Development of DTX-resistance is a common clinical problem; however, underlying mechanisms remain poorly understood. It is suggested that sustained activation of androgen-receptor (AR) signaling in CR disease [7], activation of alternative oncogenic survival pathways (such as EGFR, PI3K/Akt, MAPK/ERK) [8, 9] and overexpression of βIII-tubulin and/or drug efflux proteins [10, 11] could underlie the DTX therapeutic failure in PCa. More recently, it has also been suggested that tumor microenvironment also plays a major role in cancer chemoresistance as an ‘extrinsic *de novo*’ factor [12]. In relation to the tumor microenvironment, pathological involvement of the chemokine (C-X-C motif) ligand 12/chemokine (C-X-C motif) receptor 4 (CXCL12/CXCR4) signaling axis has been very well documented in several malignancies, including PCa [13–15]. In general, CXCR4 is activated upon binding to its sole ligand, CXCL12, which initiates a series of downstream signaling cascades responsible for downstream phenotypic responses [15, 16]. Several lines of evidence support the significance of this signaling node in PCa growth, invasion and metastasis [16-18]. Furthermore, a recent study reported that CXCL12 (produced by prostate stromal cells) protected PCa cells from DTX toxicity, an effect that was mediated through CXCR4 activation [19]. However, the mechanistic basis for this observation remained unclear.

In the present study, we have investigated the mechanism underlying chemoprotective action of CXCL12/CXCR4 signaling in PCa. Our data demonstrate that the activation of CXCL12/CXCR4 signaling counteracts DTX-induced G_2_/M phase cell cycle arrest through its effect on microtubule stability. Furthermore, we identify an important role of p21-activated kinase 4 (PAK4)-induced LIM domain kinase 1 (LIMK1) phosphorylation in mediating CXCR4 activation-induced DTX resistance. These novel findings are significant in supporting the utility of the CXCL12/CXCR4 signaling axis as a therapeutic target, and in devising improved therapeutic strategies.

## RESULTS

### Activation of CXCL12/CXCR4 signaling relieves docetaxel-induced G_2_/M phase cell cycle arrest

**Figure 1:**
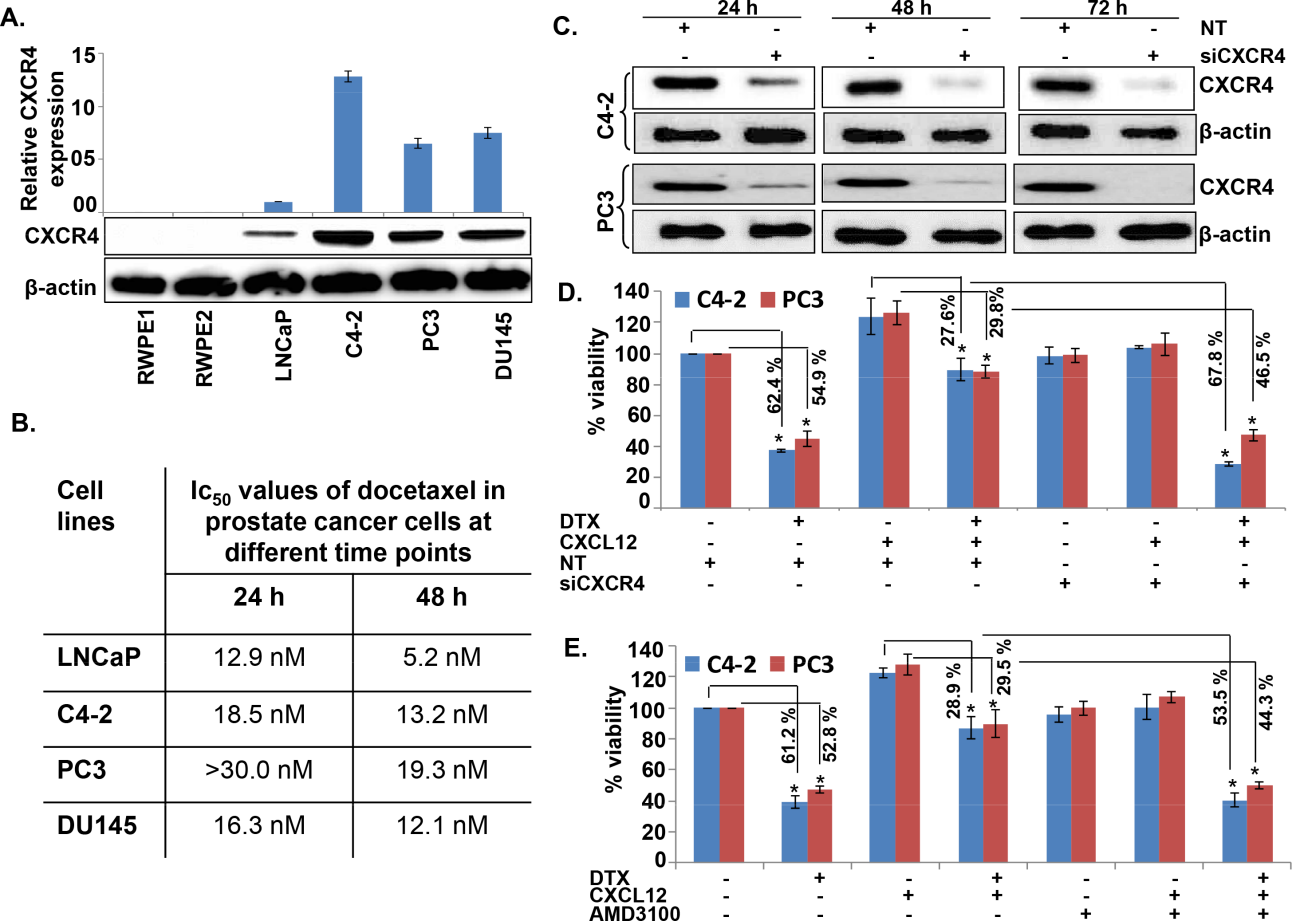
CXCR4 is overexpressed in prostate cancer cells and imparts docetaxel resistance upon CXCL12 stimulation **(A)** Total protein from normal prostate epithelial and various PCa cell lines was isolated and resolved by electrophoresis. Thereafter, expression of CXCR4 was examined by immunoblot analysis. β-actin was used as an internal control. **(B)** PCa cells were treated with the various doses of docetaxel (DTX; 0-30 nM) and the viability of cells at different time intervals (24 and 48 h) was examined by WST-1 assay and IC_50_ was calculated. **(C)** PCa cells were grown in 6-well plate and transiently transfected with non-target (NT), CXCR4-targeted siRNAs for 24–72 h. After treatment, total protein was isolated and subjected to immunoblot analysis to assess the expression of CXCR4. β-actin was used as an internal control. **(D)** C4-2 and PC3 cells were grown in 96 well plate. Thereafter, cells were treated with either CXCR4 targeting siRNAs or non-targeting control (NT-siRNA). After 24 h of transfection cells were treated with DTX (20 nM) in absence or presence of CXCL12 (100 ng/mL) and growth was monitored by WST-1 assay after 48 h. **(E)** C4-2 and PC3 cells were treated with the AMD3100 (5 μg/mL), a CXCR4 antagonist, 1 h prior to the treatment of CXCL12 followed by DTX treatment. After 48 h of treatment, viability of cells was examined by WST-1 assay. Data is presented as mean ± S.D., n = 3; *p < 0.01.

**Figure 2:**
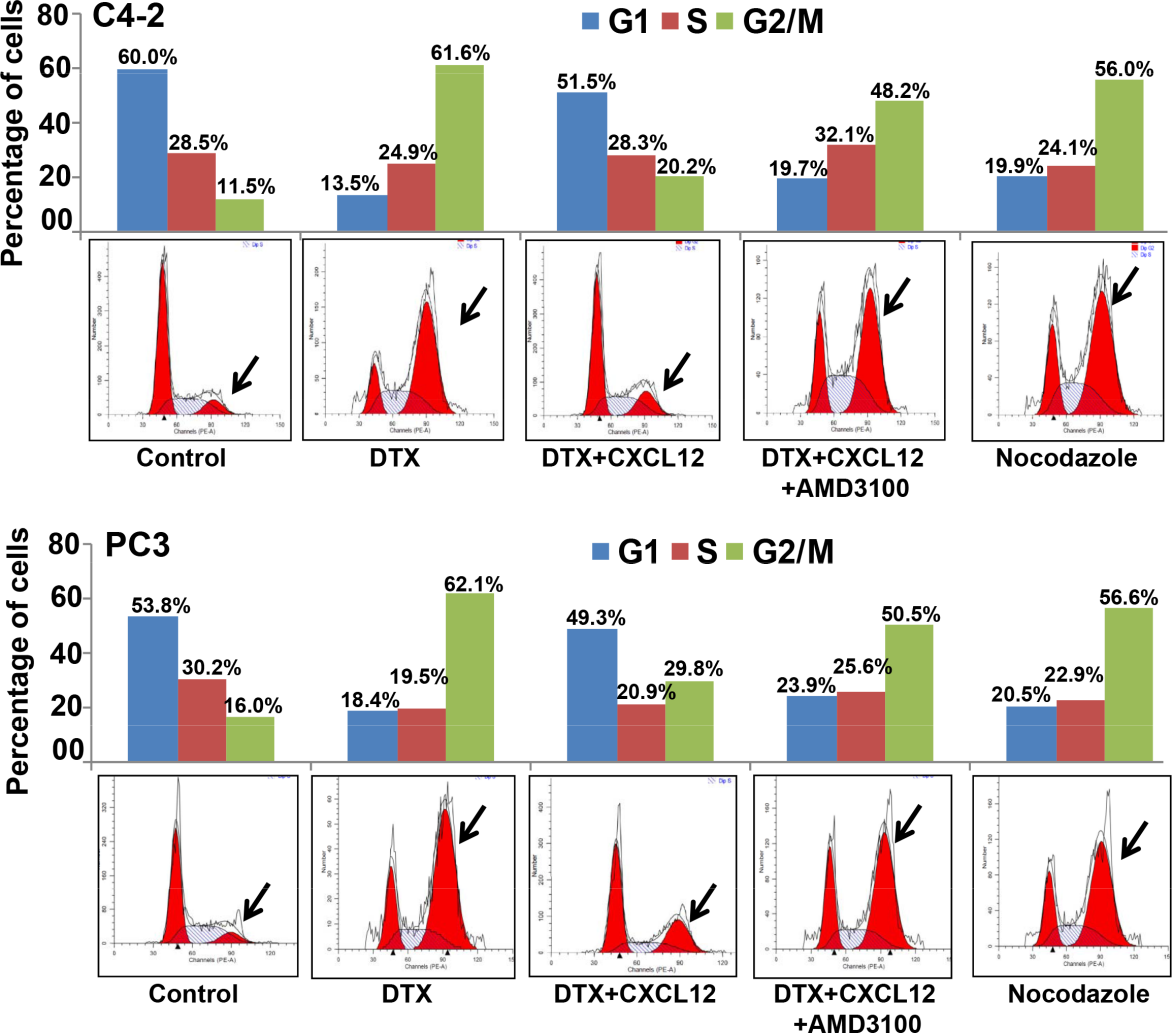
Activation of CXCL12/CXCR4 signaling relieves docetaxel-induced G_2_/M cell cycle arrest Synchronized C4-2 and PC3 cells were treated with PBS (vehicle control), docetaxel (DTX; 20 nM) alone or in combination with AMD3100 (5 μg/mL) and/or CXCL12 (100 ng/mL). After 24 h of treatment cells were fixed, stained with propidium iodide and analyzed using flow cytometry. Data show a G_2_/M phase-arrest in DTX-treated cells. CXCL12 abrogated DTX-induced G_2_/M arrest, which was reversed in the cells pre-treated with AMD3100. Nocodazole (1 μM) was used as positive control.

We first examined the expression of CXCR4 and its sole ligand, CXCL12, in a panel of PCa and normal/benign prostate epithelial (RWPE1 and 2) cell lines. An aberrant expression of CXCR4 was observed in all PCa cell lines, while no expression was detected in normal/benign prostate epithelial cells (Figure 1A). Moreover, we observed that PCa cells produced very low level of CXCL12 (range between 0.2 to 1.0 pg/ml/10^6^ cells) (data not shown). Next, we treated PCa cells with DTX (0-30 nM) for 24 and 48h and examined its toxicity. The data show that low CXCR4-expressing LNCaP cells are relatively more sensitive to DTX toxicity as compared to high CXCR4-expressing PCa cell lines (C4-2, PC3 and DU145) (Figure 1B). It should, however, be noted that the expression level of CXCR4 in PCa cell lines does not precisely correlate with docetaxel sensitivity, which could be due to the presence of additional resistance mechanisms. Nonetheless, we observe that the silencing (using specific siRNAs; Figure 1C) or inhibition (by AMD3100) of CXCR4 leads to abrogation of CXCL12-induced chemo-protection of CXCR4 in C4-2 and PC3 cells (Figure 1D and 1E). In next set of experiments, we examined whether activation of CXCL12/CXCR4 signaling axis had any effect on DTX-induced G_2_/M phase cell cycle arrest, which is an established mechanism of chemotoxic action of docetaxel [20]. C4-2 and PC3 cells were treated with DTX alone or in presence of CXCL12 and/or AMD3100, and analyzed by flow cytometry for their cell cycle distribution. Consistent with previous reports [20], the data show an arrest of cells in G_2_/M phase of cell cycle upon DTX treatment. We observe that 61.6% and 62.1% of DTX-treated C4-2 and PC3 cells, respectively, were present in G_2_/M phase as compared to 11.52% and 16.0% of control (vehicle treated) C4-2 and PC3 cells, respectively (Figure 2). Interestingly, our data demonstrate that CXCL12 treatment rescued the PCa cells from DTX-induced G_2_/M mitotic arrest, and this effect was abolished upon pretreatment of PCa cells with AMD3100 (Figure 2). Altogether, our results suggest that CXCL12/CXCR4 relieves DTX-induced G_2_/M phase cell cycle arrest in PCa cells and, thus may protect them from the cytotoxic effect of DTX.

### CXCL12/CXCR4 signaling counteracts docetaxel-induced microtubule stabilization

**Figure 3:**
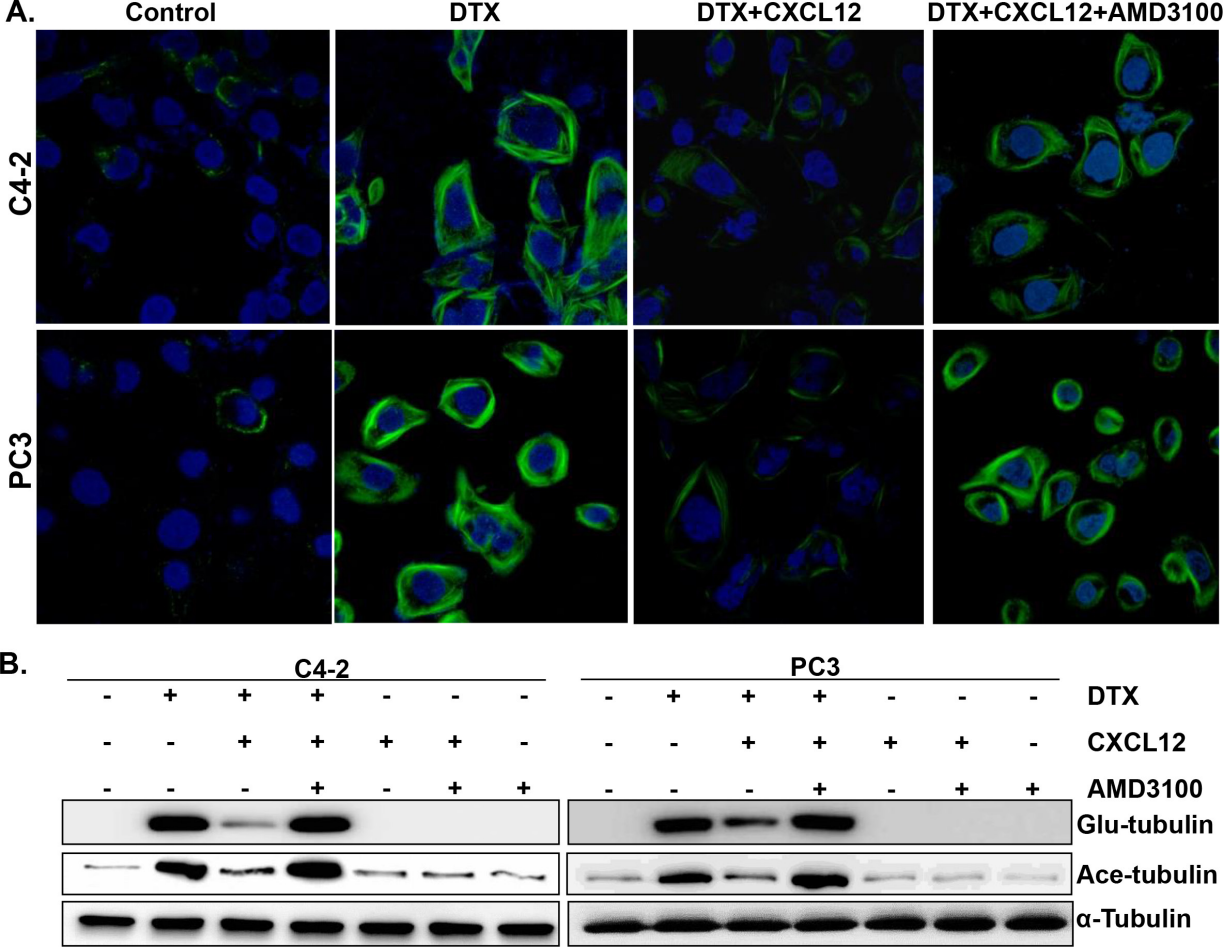
Effect of CXCL12/CXCR4 signaling on the docetaxel-induced microtubules stabilization **(A)** PCa cells (C4-2 and PC-3) were grown on glass bottom plate and treated with PBS (vehicle control), DTX alone or in combination with pre-treatment of AMD3100 (5 μg/mL) and/or CXCL12 (100 ng/mL). After 24 h of treatment, cells were fixed, stained using glu-tubulin and examined under confocal microscope. **(B)** Total protein was collected from the PCa cells treated with DTX alone or in combination with pre-treatment of AMD3100 and/or CXCL12 for 24 h. Thereafter, expression of detyrosinated (Glu), acetylated (Ace) and total α-tubulin was examined by immunoblot analysis.

DTX is a microtubule-stabilizing agent, which causes mitotic arrest following binding to polymerized tubulins and subsequent blockage of their depolymerization [6, 21]. Therefore, we investigated if CXCL12/CXCR4 signaling had an effect on DTX-induced stabilization of microtubules. For this, we performed immunofluorescence staining using an antibody against detyrosinated (glu-) tubulin, a specific marker of polymerized tubulin [22] on PCa cells either untreated or treated with DTX alone or in the presence of CXCL12. Our data demonstrate that PCa cells treated with DTX exhibit extensive formation of microtubules, an effect that is almost completely abrogated in CXCL12-treated cells (Figure 3A). Furthermore, our data show that the pre-treatment of PCa cells with a CXCR4 antagonist, AMD3100, neutralizes the effect of CXCL12 and thus restores the stabilization of microtubules (Figure 3A). To further confirm these findings, we examined the expression of tubulin polymerization markers [glu- and acetylated (ace-) tubulin] by immunoblot assay. Our data show an increased expression of both glu- and ace- tubulin in DTX-treated cells, which is suppressed in cells co-treated with CXCL12. Similarly, the pre-treatment of PCa cells with AMD3100 overrides the suppressive effect of CXCL12 (Figure 3B). Taken together, these findings demonstrate that the activation of CXCL12/CXCR4 signaling rescues the PCa cells from DTX-induced G_2_/M phase cells cycle arrest by counteracting its effect on microtubule stabilization.

### Inhibition of LIMK1 abrogates the effects of CXCL12 on docetaxel sensitivity and microtubule dynamics

**Figure 4:**
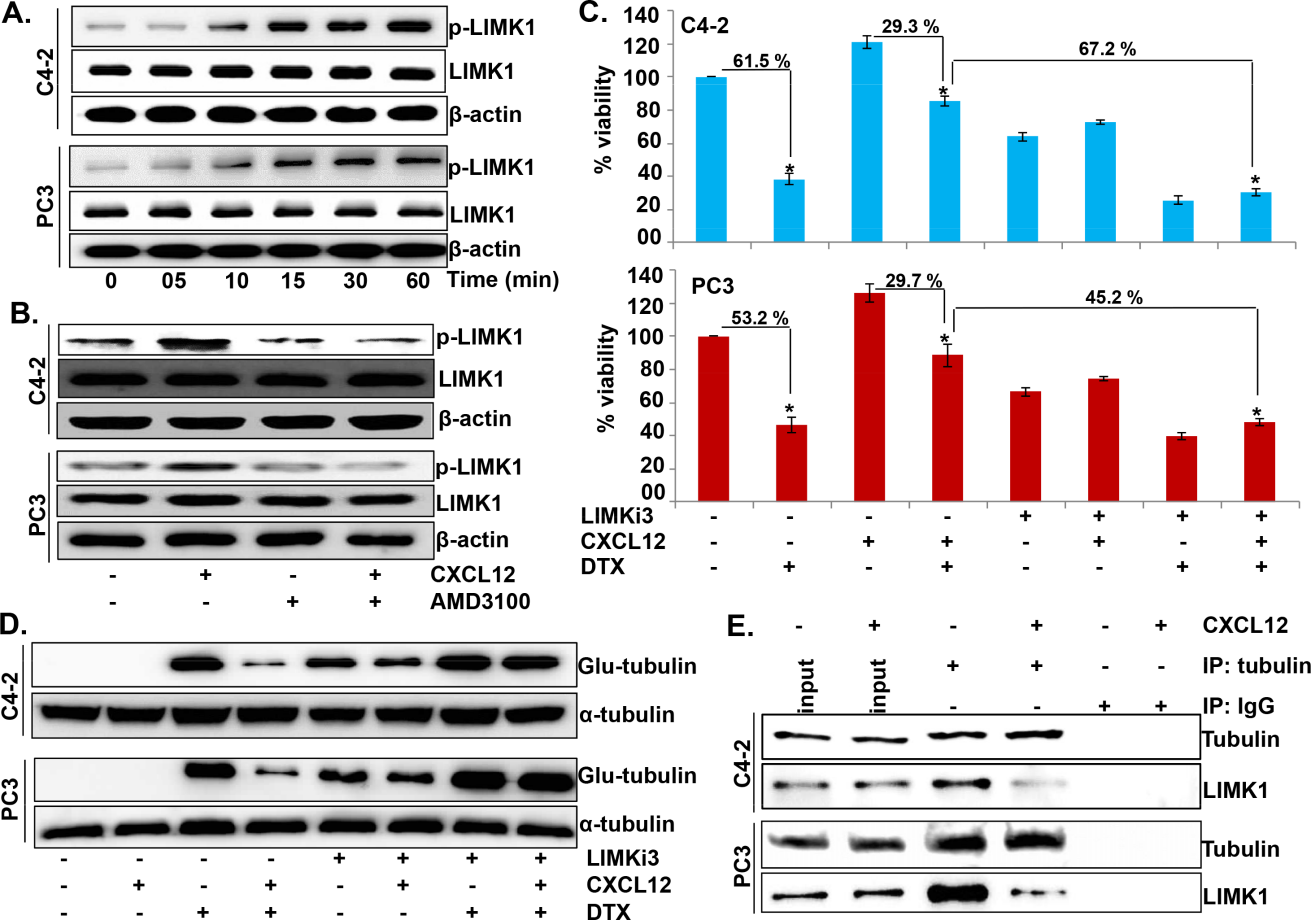
LIMK1 mediates CXCL12/CXCR4-induced destabilization of microtubules and docetaxel resistance **(A)** Cells were grown in 6-well plate and treated with CXCL12 (100 ng/mL) for various time intervals (0-60 min). Post treatment total protein was collected and expression of p-LIMK1 and total-LIMK1 was determined by immunoblot analysis. **(B)** Cells were pretreated with AMD3100 (5 μg/mL) 1 h prior to the CXCL12 treatment (100 ng/mL). After 60 min, effect on the expression of p-LIMK1 and total-LIMK1 was examined by immunoblot assay. β-actin was used as an internal control. **(C)** PCa cells were seeded in 96 well plate and treated with PBS (vehicle control), LIMKi3 (25 μM), CXCL12 (100 ng/mL) and DTX (20 nM) alone or in combination. After 48 h of treatment cell viability was measured by WST-1 assay. Data (mean ± SD; n = 3) presented as change in cell viability as compared to the control cells, *p < 0.01. **(D)** C4-2 and PC3 cells grown in 6-well plate were treated with LIMKi3 (25 μM) for 2 h, prior to the CXCL12 (100 ng/mL) and subsequent DTX (20 nM) treatment alone or in combination as described earlier. Post treatment total protein was collected and immunoblot analysis was performed to examine the expression of Glu-tubulin. α-tubulin was used as an internal control. **(E)** Immuno-precipitation assay with anti-tubulin (mouse mAb) or normal mouse IgG antibodies using the total cell lysates from control and with CXCL12-stimulated (for 30 min) PCa cells was performed. Thereafter, immuno-precipitated proteins were resolved by electrophoresis and subjected to immunoblot analysis for tubulin and LIMK1.

We next explored the molecular mechanisms by which activation of CXCL12/CXCR4 signaling counteracts DTX-induced microtubule stabilization. Our specific focus was LIMK1, which is an important downstream effector of CXCL12/CXCR4 signaling [23] and is known to regulate the stability of microtubules through direct binding [24]. Our data show that the phosphorylation of LIMK1 is increased in a time-dependent manner following CXCL12 stimulation in PCa cells (Figure 4A). Moreover, we observe that CXCL12-induced LIMK1 phosphorylation is abrogated following pretreatment with AMD3100, thus, suggesting that this effect is mediated through CXCR4 activation (Figure 4B). In next set of experiments, we treated PCa cells with LIMKi3, a LIMK1 inhibitor; prior to the treatment with CXCL12 and/or DTX and determined its effect on overall cell survival and stability of microtubules. Our data demonstrate that inhibition of LIMK1 neutralizes the rescue effect of CXCL12/CXCR4 signaling on DTX-induced cytotoxicity in both PCa cell lines (Figure 4C). The data also show that the inhibition of LIMK1 leads to abrogation of the counteracting effect of CXCL12/CXCR4 signaling on the DTX-induced microtubule stabilization (Figure 4D). Moreover, our data from immunoprecipitation assay reveal a direct interaction of LIMK1 with tubulin, which is decreased upon CXCL12 stimulation (Figure 4E). Together, these findings suggest that CXCL12/CXCR4-signaling impedes DTX-induced microtubule stabilization by promoting the phosphorylation-mediated dissociation of LIMK1 from microtubules.

### Activation of LIMK1 by CXCL12/CXCR4 signaling is mediated through PAK4

**Figure 5:**
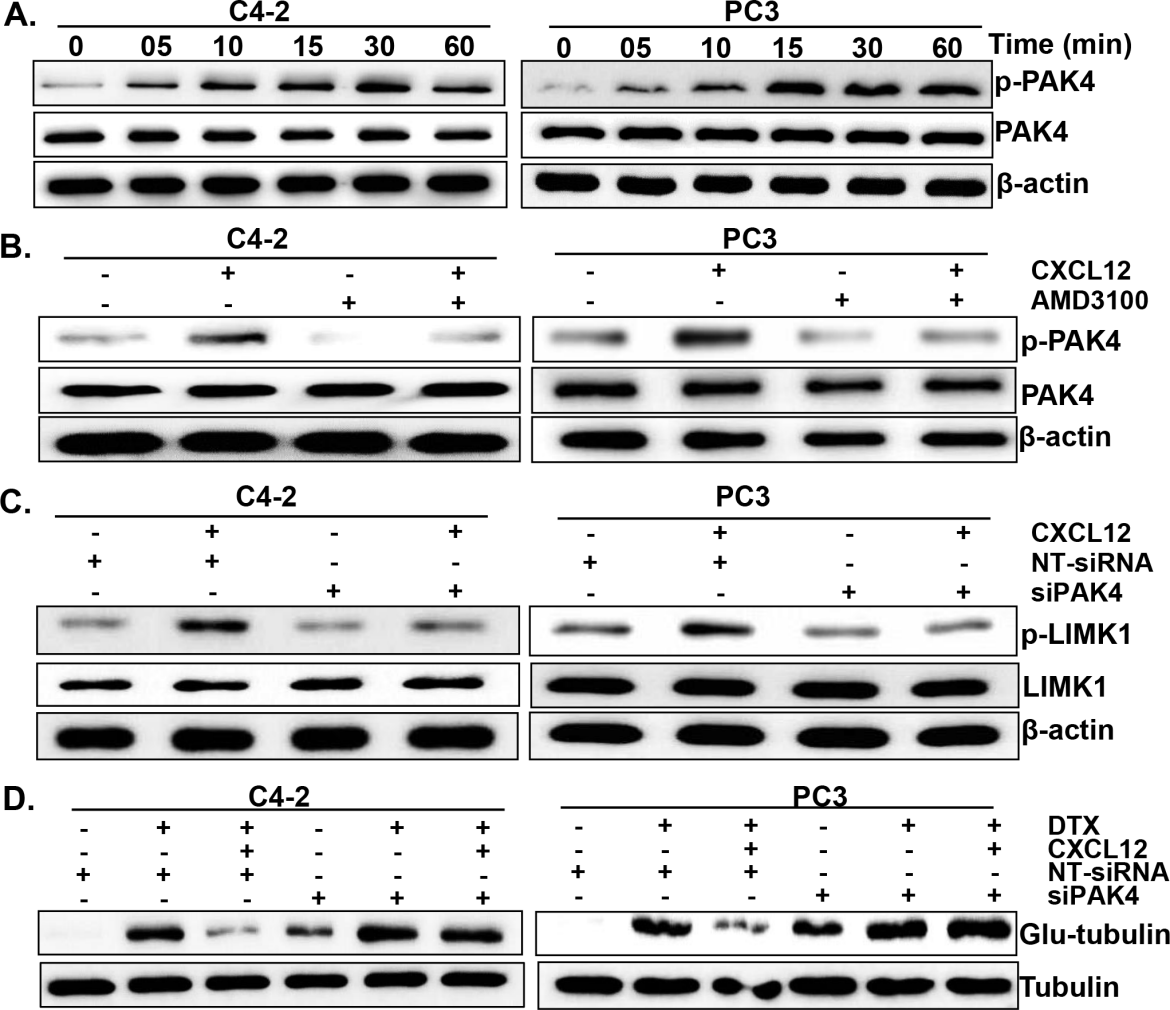
PAK4 is involved in CXCL12/CXCR4-induced LIMK1 phosphorylation **(A)** C4-2 and PC3 cells were grown in 6-well plate and treated with CXCL12 (100 ng/mL) for various time intervals (0-60 min). Thereafter, total protein was isolated, resolved, and subjected to immunoblot analysis to determine the expression of p-PAK4 and total-PAK4. **(B)** Cells were treated with AMD3100 (5 μg/mL) 1 h prior to the CXCL12 (100 ng/mL) treatment for 30 min and, effect on the expression of p-PAK4 and total-PAK4 examined by immunoblot assay. **(C)** C4-2 and PC3 cells were transfected with PAK4 targeting or non-targeting siRNAs (NT-siRNA). After 24 h of transfection, cells were treated with CXCL12 for 60 min. Post treatment, phosphorylation status of LIMK1 was determined by immunoblot assay. **(D)** PCa Cells were transfected with PAK4 specific- or NT -siRNAs prior to the DTX (20 nM) and CXCL12 (100 ng/mL) treatment. After 24 h of treatment total protein was isolated and expression of glu-tubulin was examined by immunoblot assay. β-actin was used as an internal control.

Having observed a role of LIMK1 in CXCL12/CXCR4 signaling-induced DTX resistance, we next sought to identify the protein kinase involved in its phosphorylation. For this, we focused on PAK4, which is known to cause LIMK1 phosphorylation [25]. The data show that CXCL12 treatment induces the phosphorylation of PAK4 in a time-dependent manner, which is abrogated upon pre-treatment of PCa cells with a CXCR4 antagonist (AMD3100) (Figure 5A and B). Next, we investigated if PAK4 mediates the phosphorylation of LIMK1 in response to CXCL12 treatment. For this, PAK4 was silenced using specific siRNAs prior to CXCL12 stimulation and its effect on LIMK1 phosphorylation was examined. We observed substantial silencing of PAK4 after 24 h of transfection in both the PCa cell lines treated with siPAK4 and this effect was sustained at least until 72 h of transfection (data not shown). Furthermore, we observed that the effect of CXCL12 on LIMK1 phosphorylation was abolished in PAK4-silenced PCa cells (Figure 5C). Our data also reveal that the effect of CXCL12/CXCR4 signaling on DTX-induced microtubule stabilization is nullified upon silencing of PAK4 in PCa cells (Figure 5D). Together, these data suggest that CXCL12-induced LIMK1 phosphorylation and DTX-resistance is mediated through PAK4 in PCa cells through its effect on microtubule stability.

## DISCUSSION

The present study provided mechanistic support for the chemoprotective action of CXCL12/CXCR4 signaling against DTX toxicity in PCa cells. CXCL12/CXCR4 signaling-induced DTX-resistance was caused by overriding the effect of DTX on cell cycle (G_2_/M phase arrest) due to its counteracting effect on DTX-induced microtubule stabilization. Additionally, we observed that PAK4-mediated LIMK1 activation was important in the rescue effect of CXCL12/CXCR4 signaling on DTX toxicity.

Overexpression of CXCR4 in PCa and its association with poor patients' survival has been well reported [13]. It has also been demonstrated that CXCL12/CXCR4 signaling plays an important role in the invasion and metastasis of PCa cells [16, 18], which ultimately promotes DTX-resistance in PCa [7]. Moreover, a recent study performed by Domanska et al. provides direct support to our findings, in which they have also shown the chemoprotective role of CXCL12/CXCR4 signaling in PCa cells [19]. Furthermore, Hatano et al. have reported that the DTX-mediated activation of the CXCR4, ERK1/2, and c-Myc signaling loop provides survival advantage to the PCa cells in the presence of DTX [26]. Furthermore, a role of CXCR4 signaling in the ligand-independent activation of AR has also been demonstrated, which could indirectly promote DTX-resistance by promoting cell survival [27]. Our findings further add to the list of this supporting literature by providing mechanistic support for a chemoprotective role of CXCL12/CXCR4 signaling in PCa. Our data also provide support to the notion that CXCL12/CXCR4 signaling serves as the common molecular link for the metastatic and therapy-resistant nature of the PCa cells.

We observed that the treatment with CXCL12 effectively relieved DTX-induced G_2_/M phase cell cycle arrest, an effect that was mediated through CXCR4. DTX induced G_2_/M phase arrest is mediated by inhibiting the microtubule depolymerization [20]. Microtubules are polymeric cytoskeletal structures made up of α-β-tubulin heterodimers and play an important role in the chromosomal segregation during mitosis [21, 28]. In relation to this, our data revealed that the CXCL12-mediated activation of CXCR4 counteracted DTX-induced microtubule stabilization in PCa cells. This finding is supported by an earlier study, in which a role of CXCL12/CXCR4 signaling has been demonstrated in the regulation of microtubule dynamics of immune cells [29]. Furthermore, indirect support comes from another prior report demonstrating the induction of mitotic catastrophe in ovarian cancer cells upon inhibition of CXCR4 activation [30].

Microtubules are highly dynamic structures and their intracellular dynamicity is tightly regulated by various microtubule-associated proteins (MAPs). MAPs physically interact with microtubules and promote their stabilization and/or destabilization [31]. In our study, we observed that the phosphorylation of LIMK1 was increased in PCa cells upon CXCL12 stimulation. Furthermore, data from our immunoprecipitation study revealed a physical interaction between LIMK1 and tubulin, which is decreased upon CXCL12 stimulation of PCa cells. We further observed that the inhibition of LIMK1 abolished the protective effects of CXCL12/CXCR4 signaling on DTX-induced cytotoxicity as well as on microtubule stabilization. A similar finding is reported in a recent study, where inhibition of LIMK1 in HeLa cells promoted stabilization of microtubules and subsequently led to the cell death [24]. Together, these data suggest that LIMK1 acts as a MAP in its unphosphorylated state to promote the stability of microtubules.

CXCL12/CXCR4 signaling has been shown to activate a number of downstream signaling molecules, which are involved in the regulation of growth, aggressive phenotypes and therapy resistance in cancer cells [14–16]. Here, we observed increased phosphorylation of PAK4, a member of the p21-activated kinases (PAKs) family, in CXCL12-treated PCa cells. In concordance with our data, previously Haddad et al. 2001 have also reported the increased activity of PAK4 in CXCL12 stimulated T lymphocytes [32]. To date, the exact molecular mechanism(s) of CXCL12/CXCR4-mediated PAK4 activation is not clear. However, the roles of several proteins kinases such as Rac/Cdc42, PI3K/Akt, PKC, which are the downstream effectors of CXCL12/CXCR4 signaling, have been identified in the activation of PAK4 [33]. In a recent study, Park et al. identified an association of PAK4 activation with increased tumorigenic potential and therapy resistance of PCa cells [34]. Furthermore, our data provide strong evidence that silencing of PAK4 abrogates the effects of CXCL12 on LIMK1 phosphorylation and microtubule dynamics, and thus promotes re-sensitization of the PCa cells to DTX toxicity. PAK4 interaction with LIMK1 leading to its enhanced phosphorylation has also been reported previously [25] along with demonstration of its pathological significance in PCa [35]. In the same line, our findings now shed new light on the importance of this PAK4-LIMK1 axis and establish it as an essential mediator in the chemoprotective action of the CXCL12/CXCR4 signaling pathway.

**Figure 6:**
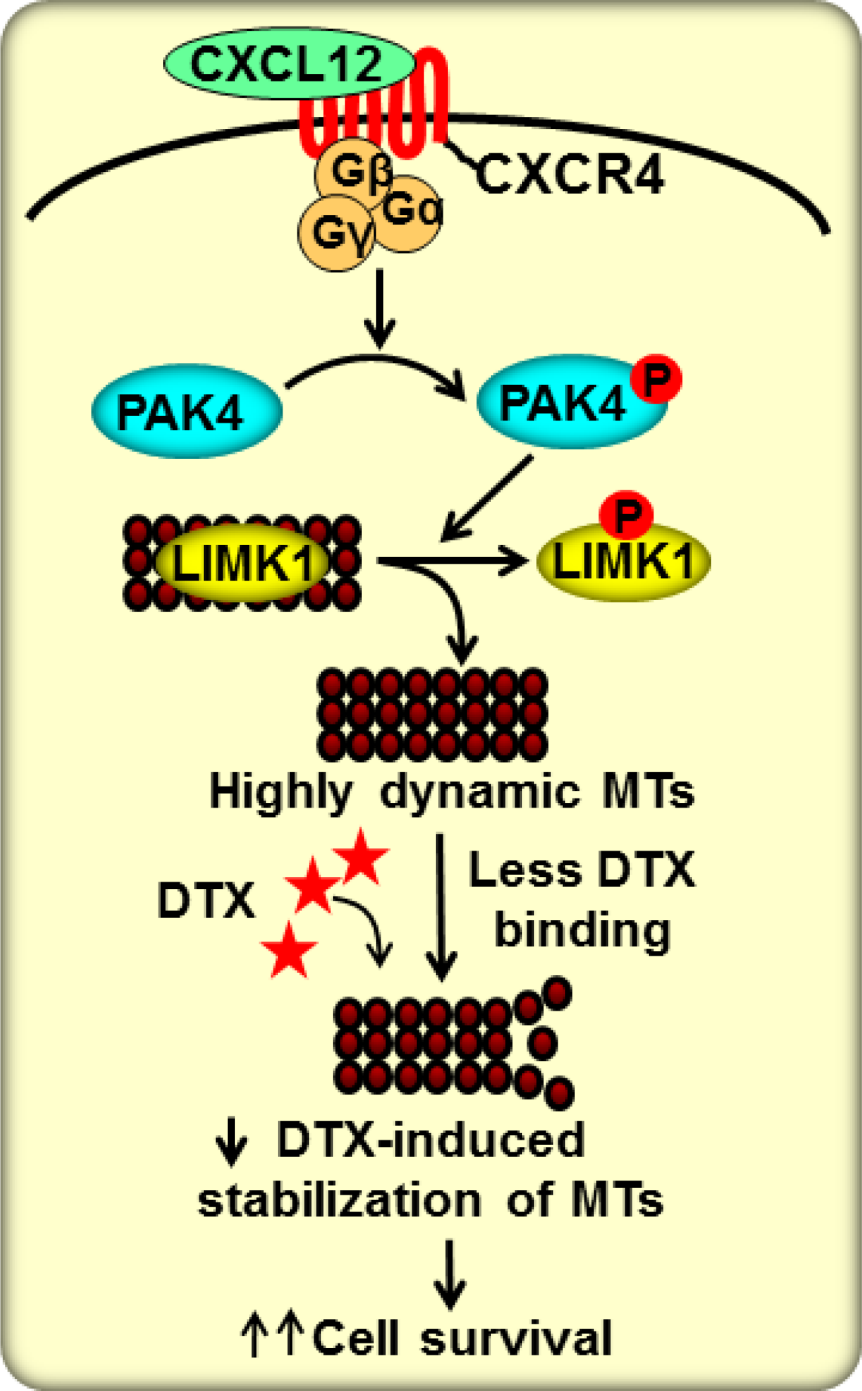
Schematic diagram for the role of CXCL12/CXCR4 and its downstream signaling in DTX resistance CXCR4 is activated upon binding to its sole ligand, CXCL12, which then leads to PAK4 phosphorylation. Activated PAK4 phosphorylates LIMK1 causing its dissociation from microtubules (MTs) and thus enhancing dynamicity of MTs. In this state, MTs are less likely to bind to DTX and consequently less susceptible to DTX-mediated stabilization. This results in suppression of anti-mitotic effect of DTX and promotes tumor cell survival.

In summary, our research findings have demonstrated an important role of the CXCL12/CXCR4 signaling axis in DTX-resistance of PCa cells through a novel mechanism (Figure 6). This new information provides additional support towards the candidacy of this signaling node as a useful target for PCa therapy.

## MATERIALS AND METHODS

### Cell lines, antibodies and siRNAs

All the cell lines used in this study were obtained, cultured and validated as described earlier [36]. Anti-CXCR4 (rabbit polyclonal), anti-detyrosinated-tubulin (glu-tubulin) and anti-acetylated-tubulin (ace-tubulin) antibodies (mouse monoclonal) were from Abcam (Cambridge, MA). Anti-phospho-LIMK1, anti-LIMK1, anti-PAK4 (rabbit polyclonal) and anti-phospho PAK4 (mouse monoclonal) antibodies were purchased from Cell Signaling Technology (Beverly, MA). Anti-α-tubulin (mouse monoclonal; for immunoprecipitation and rabbit polyclonal; for immunoblot), normal mouse IgG and all horseradish peroxidase (HRP)-conjugated secondary antibodies were from Santa Cruz Biotechnology (Santa Cruz, CA). β-actin antibody (mouse monoclonal) was from Sigma-Aldrich (St. Louis, MO). Non-target (ON-TARGET plus Non-targeting pool) and CXCR4 and PAK4 specific (ON-TARGET plus SMART pool) siRNAs and transfection reagent (DharmaFECT) were from Dharmacon (Lafayette, CO).

### Treatments and transfection

PCa cells were grown in 96- and/or 6- well plates and allowed to attain 60–70% confluence. Thereafter, cells were treated with DTX (LC labs, Woburn, MA) in the presence or absence of CXCL12 (100 ng/mL) (R&D Systems, Minneapolis, MN) as indicated in the pertinent figure legends. To dissect the role of CXCR4 and LIM kinase 1, cells were pre-incubated for 1 h with AMD3100 (CXCR4 antagonist; 5 μg/mL) (Sigma-Aldrich) and LIMKi3 (LIM kinase 1 inhibitor; 25 μM) (EMD Millipore, Billerica, MA), respectively. For the knockdown of CXCR4 and PAK4, cells were cultured in 6-well plates and transiently transfected with 50 nM of non-target or target-specific siRNAs using DharmaFECT as per the manufacturer's protocol.

### Cell growth assay

Cells were seeded in 96 well plate (3×10^3^ cells/well) a day prior to treatment. Cell growth was then examined after 24 and 48 h of treatment using WST-1 assay kit (Roche Applied Science, Indianapolis, IN) and percent growth was calculated as described previously [37].

### Immunoblot analysis

Total cells protein was extracted using NP-40 lysis buffer supplemented with protease and phosphatase inhibitors and western blotting was performed as described earlier [38]. Immunodetection was carried out using specific primary antibodies (1:1000). Thereafter, blots were incubated with respective HRP-labeled secondary antibodies (1:2500), washed and processed with ECL plus® Western Blotting detection kit (Thermo Scientific, Logan, UT) and the signal detected using an LAS-3000 image analyzer (Fuji Photo Film Co., Tokyo, Japan).

### Cell cycle analysis

Cells were synchronized by two courses of incubation (for 48 h) in serum-deprived culture media with intermittent culturing (for 24 h) in serum-containing media and then various treatments were performed. After 24 h of treatment (as indicated in figure legend), cells were washed, trypsinized and fixed with 70% ethanol overnight at 4°C. Cells were then washed with cold PBS, stained using PI/RNase kit (BD Bio Sciences, San Jose, CA) and analyzed by flow-cytometry on a BD-FACS Canto™ II (BD Bio Sciences). The percentage of cell population in various phases of cell cycle was calculated using Mod Fit LT software (Verity Software House, Topsham, ME).

### Immunofluorescence assay

PCa cells (2 × 10^3^) were grown on glass-bottom FluoroDish until sub-confluence and treated with AMD3100, CXCL12 and DTX as described above. After treatment cells were fixed in ice-cold methanol, washed, blocked and incubated with glu-tubulin antibody (1:50) for 90 min at room temperature followed by washing. Cells were then incubated with FITC-conjugated goat anti-mouse secondary antibody (1:500) for 60 min. Thereafter, cells were washed, mounted with antifade Vectashield mounting medium (Vector Labs) and observed under Nikon A1rsi Confocal Microscope System (Nikon Instruments Inc, Melville, NY).

### Immunoprecipitation assay

Total protein from the control and CXCL12-treated PCa cells was collected and estimated using DC Protein Assay Kit (Bio-Rad, Hercules, CA). Subsequently, protein lysates were incubated overnight at 4°C with anti-α-tubulin (mouse mAb) or normal mouse IgG antibodies (100:1) followed by incubation with Protein A agarose beads (Thermo Scientific, Rockford, IL) for next 2–3 h. Resulting antigen-antibody complex was centrifuged at low speed (2500 g), washed and eluted. Thereafter, whole cell lysate (input) and immunoprecipitated proteins were subjected to electrophoresis and immunoprobed for α-tubulin and LIMK1.

### Statistical analysis

All the experiments were performed at least three times. The data obtained were expressed as ‘mean’ standard deviation' and subjected to unpaired two tailed Student's t-test. A value of p < 0.05 was considered as significant.
